# Intermediate and Long-term Outcomes of Survivors of Acute Kidney Injury Episodes: A Large Population-Based Cohort Study

**DOI:** 10.1053/j.ajkd.2016.05.018

**Published:** 2017-01

**Authors:** Simon Sawhney, Angharad Marks, Nick Fluck, Adeera Levin, Gordon Prescott, Corri Black

**Affiliations:** 1 University of Aberdeen Applied Renal Research Collaboration, Aberdeen, Scotland; 2 NHS Grampian, Aberdeen, Scotland; 3 Farr Institute@Scotland, Aberdeen, Scotland; 4 University of British Columbia, Vancouver, Canada

**Keywords:** Acute kidney injury (AKI), chronic kidney disease (CKD), baseline kidney function, survival, mortality, epidemiology, outcomes, prognosis, AKI recovery, acute on chronic kidney disease

## Abstract

**Background:**

The long-term prognosis after acute kidney injury (AKI) is variable. It is unclear how the prognosis of AKI and its relationship to prognostic factors (baseline kidney function, AKI severity, prior AKI episodes, and recovery of kidney function) change as follow-up progresses.

**Study Design:**

Observational cohort study.

**Setting & Participants:**

The Grampian Laboratory Outcomes Morbidity and Mortality Study II (GLOMMS-II) is a large regional population cohort with complete serial biochemistry and outcome data capture through data linkage. From GLOMMS-II, we followed up 17,630 patients hospitalized in 2003 through to 2013.

**Predictors:**

AKI identified using KDIGO (Kidney Disease: Improving Global Outcomes) serum creatinine criteria, characterized by baseline kidney function (estimated glomerular filtration rate [eGFR] ≥ 60, 45-59, 30-44, and <30 mL/min/1.73 m^2^), AKI severity (KDIGO stage), 90-day recovery of kidney function, and prior AKI episodes.

**Outcomes:**

Intermediate- (30-364 days) and long-term (1-10 years) mortality and long-term renal replacement therapy.

**Measurements:**

Poisson regression in time discrete intervals. Multivariable Cox regression for those at risk in the intermediate and long term, adjusted for age, sex, baseline comorbid conditions, and acute admission circumstances.

**Results:**

Of 17,630 patients followed up for a median of 9.0 years, 9,251 died. Estimated incidences of hospital AKI were 8.4% and 17.6% for baseline eGFRs ≥ 60 and <60 mL/min/1.73 m^2^, respectively. Intermediate-term (30-364 days) adjusted mortality HRs for AKI versus no AKI were 2.48 (95% CI, 2.15-2.88), 2.50 (95% CI, 2.04-3.06), 1.90 (95% CI, 1.51-2.39), and 1.63 (95% CI, 1.20-2.22) for eGFRs ≥ 60, 45 to 59, 30 to 44, and <30 mL/min/1.73 m^2^, respectively. Among 1-year survivors, long-term HRs were attenuated: 1.44 (95% CI, 1.31-1.58), 1.25 (95% CI, 1.09-1.43), 1.21 (95% CI, 1.03-1.42), and 1.08 (95% CI, 0.85-1.36), respectively. The excess long-term hazards in AKI were lower for lower baseline eGFRs (*P* for interaction = 0.01).

**Limitations:**

Nonprotocolized observational data. No adjustment for albuminuria.

**Conclusions:**

The prognostic importance of a discrete AKI episode lessens over time. Baseline kidney function is of greater long-term importance.

Editorial, p. 3

Acute kidney injury (AKI) occurs in 1 in 7 hospital admissions.^1^ Even those with small increases in creatinine levels have 4-fold greater hospital mortality than those with no creatinine level increase.^2^ Overall, for those who survive to hospital discharge, the prognosis remains poor. However, although 2 previous systematic reviews reported long-term increased mortality and long-term renal replacement therapy (RRT) after AKI (vs no AKI), there was substantial heterogeneity in both outcomes (*I*^2^ > 85%).^3^, ^4^ To discuss risk and plan care for individual patients requires a better understanding of what drives variation, which patients are at elevated risk, and how long risk remains elevated.^5^

Clinical guidelines recognize that the long-term prognostic factors for AKI are poorly understood.^6^, ^7^ The KDIGO (Kidney Disease: Improving Global Outcomes) guideline advocates follow-up of all patients with AKI,^6^ and UK guidelines recommend surveillance for at least 2 to 3 years.^8^ Monitoring of all patients may be prudent, but in clinical practice, some patients are prioritized over others, only a minority see a nephrologist, and follow-up may be brief.^9^, ^10^ Without more detailed prognostic studies, it is difficult for clinicians to communicate individual risks and prioritize high-risk patients for an appropriate duration.

Potential drivers of variation in AKI prognosis include the limited availability of pre-AKI (baseline) data in previous AKI definitions, differences in the severity of AKI, level of baseline kidney function, degree of subsequent recovery of kidney function, and variation in the prognostic role of AKI as follow-up time progresses.^3^, ^4^, ^11^, ^12^, ^13^ These factors are potentially quantifiable in all patients with AKI, but have not been systematically explored in any one study. In particular, the changing prognostic role of AKI at different levels of baseline function and at different follow-up times has received little attention, and the relevance of prior AKI episodes has not previously been studied.^11^ Furthermore, the time at which recovery of kidney function is assessed has varied in previous studies, and it is unclear how often this will have reclassified patients.

The Grampian Laboratory Outcomes Morbidity and Mortality Study II (GLOMMS-II) is a population cohort linking national and regional data sources in a single UK health authority. Uniquely, all biochemistry data are obtained by a single laboratory service regardless of clinical location (inpatient, outpatient, and community), thus minimizing the loss of baseline and follow-up data. We have previously exploited GLOMMS-II to study different approaches to using kidney function data to define AKI in clinical practice and prognostic research.^14^, ^15^

To guide decisions and discussions with patients about the implications of an AKI episode, we have used GLOMMS-II to study how the prognosis after isolated AKI episodes changes during the course of a long follow-up from intermediate (30-364 days) to long term (1-10 years). We hypothesized that the adverse prognosis of AKI would lessen over time and the role of AKI prognostic factors (baseline, severity, and prior AKI episodes) would change as follow-up progressed.

## Methods

### Population

The GLOMMS-II was developed through novel data linkage of regional biochemistry results (1999-2009) to hospital episode data, the local renal information management system, and the Scottish Renal Registry for long-term RRT, morbidity, and outcomes.^14^, ^16^ It includes all patients with abnormal kidney function test results (estimated glomerular filtration rate [eGFR] < 60 mL/min/1.73 m^2^) and a 20% sample of those with normal kidney function. It has been extensively used in renal research, including AKI.^14^, ^16^, ^17^, ^18^ All serum creatinine measurements are isotope-dilution mass spectrometry−aligned and processed by a single biochemistry service. Data linkage enables population follow-up without formal patient recruitment, which minimizes selection biases. Information Services Division (ISD) Scotland refreshed the linkages using the community health index, a unique identifier for all residents in Scotland, to connect each AKI episode to individual hospital admissions. There were no patients without a community health index indexed in the ISD population “spine,” meaning that all records were linkable. The ISD reports precision of 99.9% for record linkages.^19^ We obtained approval from the Privacy Advisory Committee (study number XRB14137) and the Regional Ethics Committee (reference 14/NW/1371), which waived the requirement for informed consent for this study. Data were hosted and managed by Grampian Data Safe Haven.^20^

### Exposure: Admission With AKI in 2003

The exposure was the first hospital admission with AKI in 2003. We excluded those with AKI who were not admitted to the hospital within 7 days. This was to ensure that both the exposed and comparator groups contained only admitted patients and because KDIGO-based AKI criteria perform differently in patients who are not admitted to the hospital.^21^ We defined AKI using a modification of the National Health Service (NHS) England AKI “e-alert” algorithm, derived from the KDIGO AKI definition (Box 1).^22^ The first result meeting AKI criteria was recorded as the start of an AKI episode, and the corresponding “look-back” result from the criteria was used as the baseline.

**Supplementary Figure S1:**
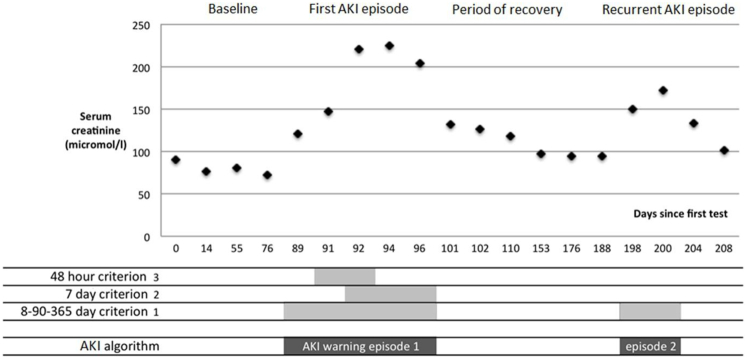
Illustration of modified version of NHS England e-alert algorithm used in this study.

We have demonstrated the performance of the NHS England algorithm elsewhere,^14^ but because it does not organize blood tests into discrete AKI episodes, we developed a modified version that was capable of distinguishing AKI episode severity and prior and subsequent AKI episodes. The original algorithm uses an 8- to 365-day look-back to estimate the median baseline creatinine level, but a prior AKI episode could result in a falsely high estimate of baseline from tests in the past year leading to underdetection of recurrent AKI.^23^ Therefore, as illustrated in Fig S1 (available as online supplementary material), we modified the algorithm criteria to a 1.5-fold or greater increase from median creatinine level within 8 to 90 days in those with an available test, and further look-back to 365 days only in those without a more recent test (criterion 1, Box 1). To ensure clinical relevance for those using the original NHS England algorithm, we compared diagnostic agreement and 30-day mortality in a sensitivity analysis.

### Definitions for AKI Severity, Prior AKI Episodes, and Recovery of Kidney Function

Definitions are summarized in Box 1. We determined AKI severity stages 1 to 3 using the peak creatinine level in the AKI episode relative to the baseline creatinine level. We counted the number of prior AKI episodes that occurred in the preceding 91 to 1,095 days (ie, 3 years). Given the varying definitions of recovery and the KDIGO recommendation of reassessment at 90 days,^6^, ^13^, ^24^, ^25^, ^26^ we determined how an earlier assessment (7 days) compared to a later assessment (90 days) with regard to recovery status and completeness of repeat testing. Because our interest was in the classification of those with complete return to baseline,^27^ those receiving RRT were classed as nonrecovery rather than grouped separately.

### Comparator: Admission Without AKI in 2003

Comparators were patients admitted to the hospital in 2003, who had a blood test for kidney function during admission, and did not have AKI. To ensure that they had the opportunity to develop AKI in 2003, the last admission in 2003 was used. We used their hospital admission creatinine as baseline.

### Outcomes

Outcomes were mortality and long-term RRT (dialysis or transplantation).

### Follow-up

Follow-up was from the date of initial hospital admission to 2013. Follow-up was through data linkage to the national register rather than direct patient contact. Migration from Grampian to beyond Scotland was negligible for the period and age-mix of the cohort.^28^ Those not registered as dead during follow-up were therefore assumed to be still alive.

### Covariates of Interest

We used *International Classification of Diseases, Tenth Revision* codes for Charlson comorbid conditions from the 5 years prior to admission as previously described and validated.^29^, ^30^ We also determined diagnostic categories (eg, circulatory system and respiratory system) related to the acute admission by using the admission categories grouped and recorded in *International Classification of Diseases, Tenth Revision*, as previously utilized elsewhere^2^. We used the CKD-EPI (Chronic Kidney Disease Epidemiology Collaboration) creatinine equation to describe baseline kidney function in 4 eGFR groups^31^: normal, ≥60; mild decrease, 45 to 59; moderate decrease, 30 to 44; and severe decrease, <30 mL/min/1.73 m^2^. We did not include measures of proteinuria because only a minority of the population had been tested. We also recorded whether the admission was an emergency readmission and the specialty involved during the hospital admission with the following priority when more than 1 was involved: critical care, surgical, care of the elderly, medical, and “other” (eg, obstetrics and psychiatry).

### Analysis

We reported the incidence of hospital AKI in those with and without baseline decreased kidney function. Due to cohort sampling, those with normal baseline function and no AKI were under-represented. This means that the incidence of AKI among those admitted with normal baseline function cannot be taken directly from the data presented in Table 1 because this would overestimate its occurrence. Therefore, the incidence of AKI was calculated directly for those with baseline decreased kidney function and estimated by multiplying out the sampled fraction for those with normal baseline function. We described overall characteristics and outcomes stratified by baseline kidney function, AKI severity stage, and history of prior AKI episodes. We compared patient recovery of kidney function status at 7 and 90 days, including the proportion of patients who improved, deteriorated, and died. We reported crude mortality and long-term RRT and plotted 10-year mortality by AKI stage using 1 − Kaplan-Meier curves. We assessed mortality in intervals of 0 to 30 days, 31 to 90 days, 91 to 182 days, 183 to 364 days, 1 to less than 3 years, and 3 to 5 years. Within each interval, we computed mortality rates by AKI stage stratified by baseline eGFR group. Using Poisson regression, we calculated age- and sex-adjusted mortality rate ratios (RRs) within each period by AKI stage and prior AKI episodes. We then determined long-term (1-10 years) mortality among those alive at 1 year using multivariable Cox regression adjusted for age, sex, and covariates of interest. Based on previous literature, we included an interaction term between baseline eGFR and AKI on mortality.^11^, ^32^ In a series of sensitivity analyses, we also calculated hazard ratios (HRs) for intermediate outcomes (30-364 days), and when analysis was restricted to those younger than 75 years, to patients with AKI without complete recovery to baseline, and those who also had prior AKI episodes. All analyses were conducted using Stata/SE 13.0 (StataCorp LP).

## Results

### Cohort Description

**Figure 1:**
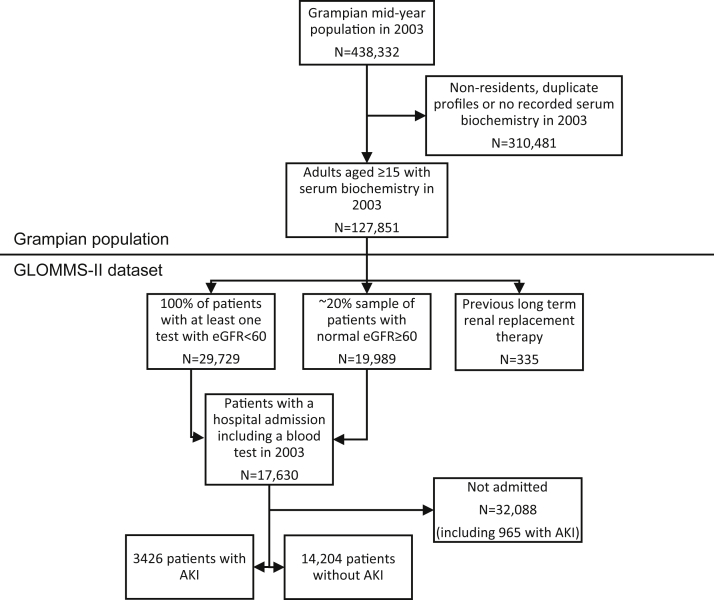
Flow chart of study population from GLOMMS-II (Grampian Laboratory Morbidity and Mortality Study II). Abbreviations: AKI, acute kidney injury; eGFR, estimated glomerular filtration rate.

The GLOMMS-II is outlined in Fig 1. Of 17,630 hospitalized patients, 3,426 (19.6%) had AKI. The estimated incidence of AKI in the hospital was 17.6% among patients with baseline decreased kidney function and 8.4% among patients with normal baseline function. The 10-year observation period extended from 2003 until 2013 and comprised 114,696 person-years (median follow-up, 9.0 years).

### Characteristics of Patients With and Without AKI

Patient characteristics for those with and without AKI are described in Table 1. Data are stratified by baseline kidney function to allow for population sampling. The left panel describes patients with and without AKI further stratified by AKI severity. Of those with normal baseline function, patients with AKI were older, received more critical care, and had more comorbid conditions than patients without AKI, but age and comorbid conditions varied little with AKI severity. The pattern was similar for those with baseline decreased kidney function, though all groups were older and had more comorbid conditions. Additional data for patient characteristics in eGFR subgroups are provided in Table S1.

The Table 1 right panel focuses on the 3,426 patients with AKI, by history of prior AKI episodes. There were 688 (20.1%) patients who had prior AKI episodes, of which 38.4% had occurred within the last 1 year. Patients with prior AKI episodes had more comorbid conditions than those without prior AKI (eg, congestive heart failure and diabetes).

**Supplementary Figure S2:**
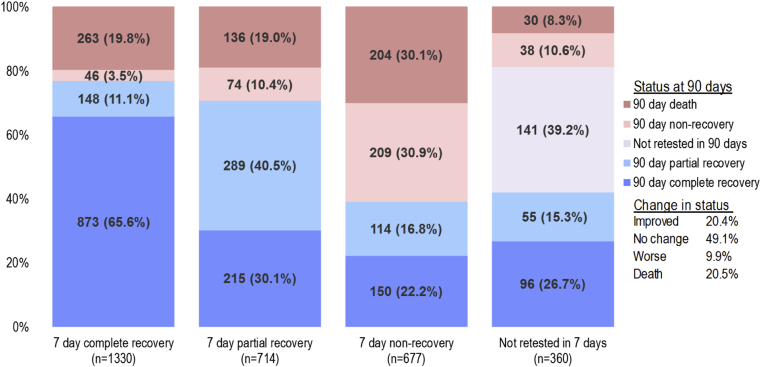
Comparison of recovery-of-kidney-function status at 7 and 90 days.

As reported in Fig S2, the recovery status of many patients with AKI changed between 7 and 90 days. Of the 3,081 of 3,426 patients with AKI still alive at 7 days, by 90 days, 20.4% had improved, 9.9% had deteriorated, 20.5% had died, and 49.1% were unchanged from 7 days. At 7 days, a substantial proportion (360 of 3,081 [11.7%]) had also not yet had a repeat blood test. For these reasons, subsequent analysis used recovery of kidney function at 90 days.

### Mortality and RRT Outcomes

Tables 2 and S2 summarize crude mortality and long-term RRT. Irrespective of baseline function, mortality was higher for AKI versus no AKI, even at 10 years (Table 2). In those with normal baseline kidney function, long-term RRT was rare, irrespective of AKI episode, severity, or duration (Table S2). In those with baseline decreased kidney function, long-term RRT was most frequent in patients with AKI stage 3. It was also more frequent in those with prior AKI episodes. Further data for long-term RRT in eGFR subgroups are available in Table S3.

### Effect of AKI on Mortality Over Intermediate- and Long-term Intervals

**Figure 2:**
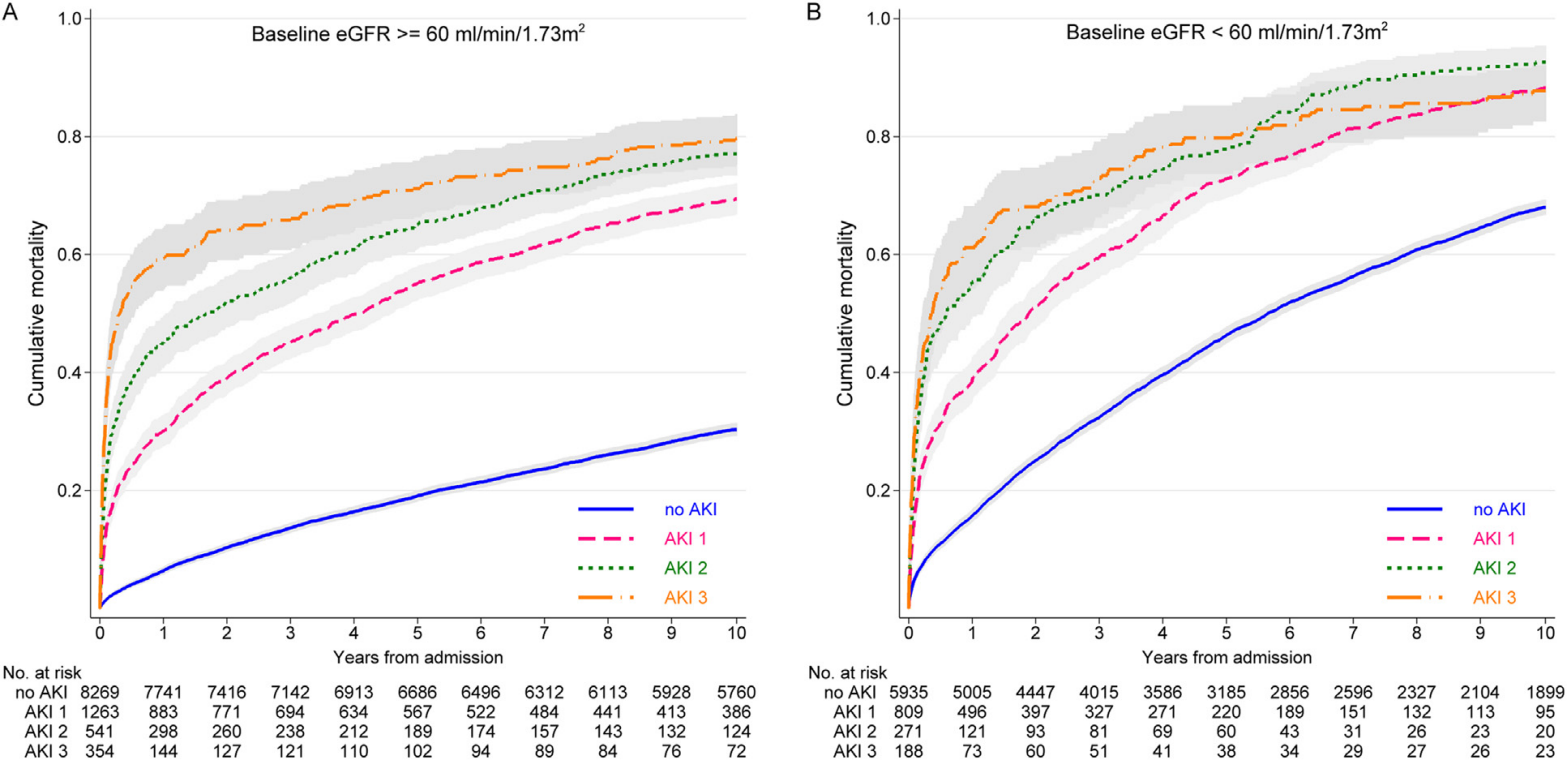
Cumulative mortality by acute kidney injury (AKI) stage (1-3 denote severity stage), stratified by baseline kidney function. Abbreviation: eGFR, estimated glomerular filtration rate (mL/min/1.73 m^2^).

**Figure 3:**
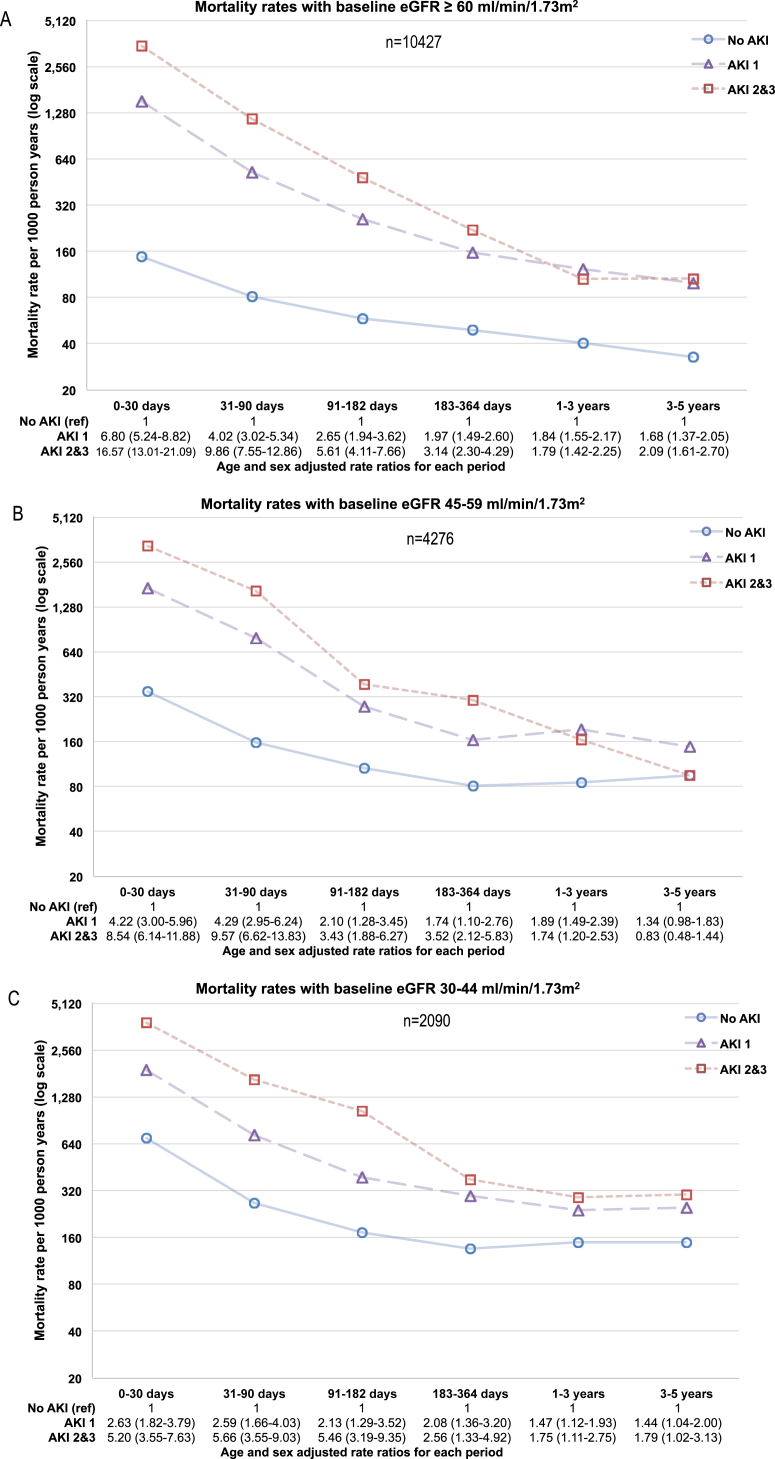

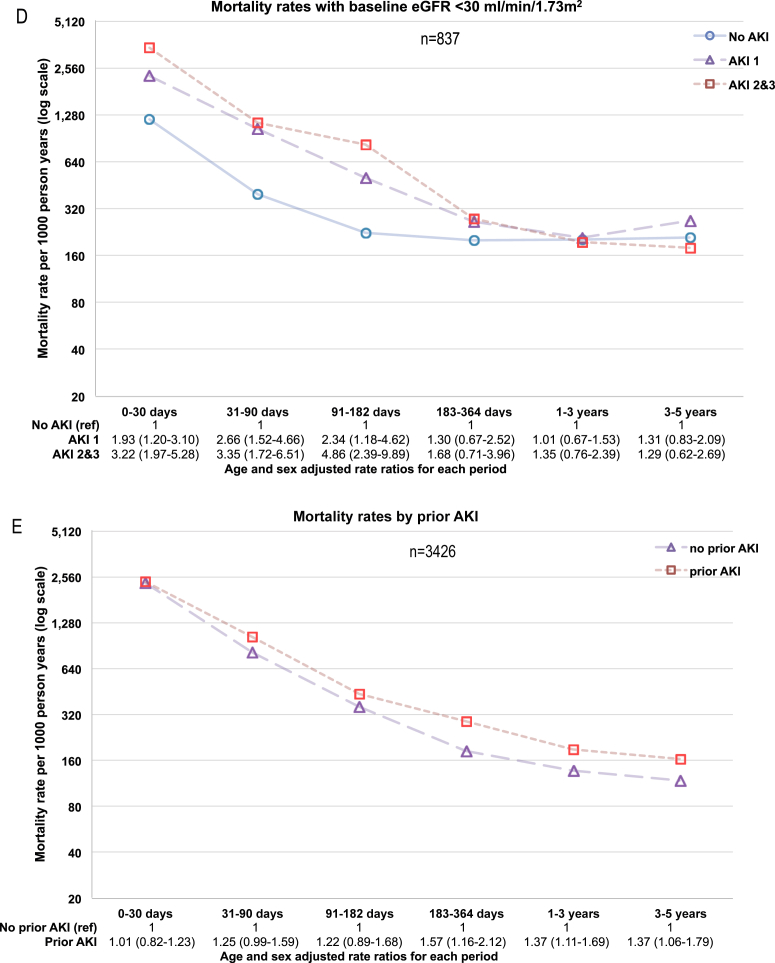
Mortality rates and age- and sex-adjusted rate ratios by (A-D) baseline estimated glomerular filtration rate (eGFR) group and (E) prior acute kidney injury (AKI; 1-3 denote severity stage). Abbreviation: ref, reference group.

Regardless of baseline eGFR, the more severe the AKI, the greater the initial mortality (Fig 2). However, after 1 year, the mortality curves no longer diverged. Figure 3 describes the change in mortality rates over the first 5 years in greater detail. Figure 3A to D describes mortality rates in patients at risk in each of 6 intervals (0-30 days, 31-90 days, 91-182 days, 183-364 days, 1-<3 years, and 3-5 years) by AKI severity and stratified by baseline eGFR. Figure 3E describes mortality rates by prior AKI episodes. Age- and sex-adjusted RRs and 95% confidence intervals (CIs) are reported. At all levels of baseline eGFR, early mortality rates were higher in those with AKI than without AKI and highest in those with AKI stages 2 to 3. The early (0-30 days) mortality RRs for AKI (vs no AKI) were greater in those with higher baseline eGFRs: RRs of 6.80 (95% CI, 5.24-8.82), 4.22 (95% CI, 3.00-5.96), 2.63 (95% CI, 1.82-3.79), and 1.93 (95% CI, 1.20-3.10) for AKI stage 1 at eGFRs ≥ 60, 45 to 59, 30 to 44, and <30 mL/min/1.73 m^2^, respectively. In the same eGFR subgroups, the association of AKI (vs no AKI) with mortality also diminished over time: RRs at 1 year of 1.84 (95% CI, 1.55-2.17), 1.89 (95% CI, 1.49-2.39), 1.47 (95% CI, 1.12-1.93), and 1.01 (95% CI, 0.67-1.53), respectively. Those with prior AKI episodes (vs no prior episodes) had similar early mortality, but greater mortality from 6 months onward.

### Adjusted Long-term Mortality

Table 3 describes the relationship between AKI and long-term mortality (conditional on surviving 1 year) in adjusted models (age, sex, baseline comorbid conditions, and acute circumstances). There was effect modification by baseline eGFR (*P* for interaction = 0.01). Among 1-year survivors, the adjusted mortality HRs for AKI (vs no AKI) were lower at lower baseline eGFRs: HRs of 1.44 (95% CI, 1.31-1.58), 1.25 (95% CI, 1.09-1.43), 1.21 (95% CI, 1.03-1.42), and 1.08 (95% CI, 0.85-1.36) for eGFRs ≥ 60, 45 to 59, 30 to 44, and <30 mL/min/1.73 m^2^, respectively. As demonstrated by a comparison to those who had no AKI and baseline eGFRs ≥ 60 mL/min/1.73 m^2^ (Table 3), AKI made little difference among those with lower baseline eGFRs because their mortality was high irrespective of AKI.

### Sensitivity Analyses

In a series of sensitivity analyses for mortality outcomes (Table S4), adjusted mortality HRs were higher when calculated for intermediate outcomes (30-364 days): 2.48 (95% CI, 2.15-2.88), 2.50 (95% CI, 2.04-3.06), 1.90 (95% CI, 1.51-2.39), and 1.63 (95% CI, 1.20-2.22) for eGFRs ≥ 60, 45 to 59, 30 to 44, and <30 mL/min/1.73 m^2^, respectively. HRs were similar when limited to those younger than 75 years or when limited to patients with AKI without complete recovery to baseline at 90 days. Although those with prior AKI episodes had higher mortality HRs, this could be explained by adjusting for comorbid conditions and acute circumstances.

In this study, we modified the widely used NHS England AKI warning algorithm to enable the grouping of blood tests into discrete AKI episodes and the identification of prior AKI episodes. There was substantial overlap with the original algorithm with 99.2% agreement in diagnosis and a κ statistic of 0.95. Moreover, 30-day AKI mortality differed by only 0.2% (20.1% modified algorithm, 19.9% original algorithm).

## Discussion

The relationship between a discrete episode of AKI and outcome is complex. This large population study with long follow-up demonstrates the diminishing prognostic role of AKI over time, with risk only modestly increased among 1-year survivors, especially those with baseline decreased kidney function. Both intermediate- and long-term mortality with AKI were modified by baseline kidney function, with the greatest association of AKI with mortality in those with normal baseline function. In contrast, although AKI was not associated with significantly increased long-term mortality among those with baseline eGFRs < 30 mL/min/1.73 m^2^, this group of patients still had the highest absolute mortality risk. Over time, there were also different prognostic roles for AKI severity and prior AKI. Severe AKI had greater short-term mortality, but AKI severity was less relevant as follow-up progressed. Patients with prior AKI episodes had greater long-term mortality, although this could be explained by comorbid conditions and acute circumstances. Finally, although patients with AKI received more long-term RRT, RRT was a rare outcome in the absence of baseline decreased kidney function.

This analysis is consistent with and extends previous work. Previous studies have also noted effect modification by baseline function^12^, ^32^, ^33^ and a diminishing role of AKI after hospital discharge.^34^, ^35^ This analysis now shows that the diminishing role of AKI continues for up to 1 year after AKI and is present at all levels of baseline kidney function. The description of prior AKI as a prognostic factor is also novel and important. One in 5 patients with AKI had prior AKI episodes, with worse long-term mortality. Prior AKI was particularly common in those with heart failure, which is consistent with a recent study that reported increased recurrent AKI in heart failure but did not describe the long-term consequences.^36^ Finally, the 2-fold increased incidence of hospital AKI in those with baseline decreased kidney function (vs normal baseline function) is in agreement with recent reports elsewhere.^37^

A strength of this analysis is our use of a large unselected population all served by a single biochemistry service, and laboratory data capture all patients throughout the time course of observation, pre– and post–hospital admission. The unique situation ensures the completeness of the data over a prolonged follow-up and overcomes the shortcomings of other studies wherein selective laboratory testing or access to tests would lead to missing baseline or follow-up data. We also based our AKI definition on an existing e-alert algorithm. Our analysis is therefore particularly relevant for clinicians who are evaluating patients with AKI e-alerts or are developing similar AKI e-alert systems. However, we recognize and have reported elsewhere that some misclassification of CKD occurs.^15^

A relative limitation, which may be also a strength for real-world generalizability, is the fact that laboratory data were not protocolized with respect to intervals of data collection. Blood testing performed at clinical discretion rather than in standardized testing intervals introduces an ascertainment bias. This is not unique to our study and reflects real-life practice, but it is possible that we have missed some AKI cases. In addition, long-term RRT was a rare outcome. Although this prevented more detailed subgroup analyses, the paucity of long-term RRT among people without baseline decreased kidney function was still striking.

A key message of this study is that risk after AKI is not static, but changes over the course of a long follow-up. The diminishing association of an AKI episode with mortality is accompanied by greater importance of long-term factors (baseline kidney function and history of prior AKI episodes) over acute factors (AKI severity). Future work should now explore whether these factors at different time points can be used to form prognostic prediction tools for use in clinical practice.

## Figures and Tables

**Table 1:** Patient Characteristics by Baseline Kidney Function and AKI Severity, and by Prior AKI

	Of 17,630 Patients in the Entire Cohort by Baseline eGFR and AKI Severity										Of 3,426 AKI Patients Only		
	Normal Baseline Kidney Function: eGFR ≥ 60 mL/min/1.73 m^2^					Baseline Decreased Kidney Function: eGFR < 60 mL/min/1.73 m^2^							
	No AKI (n = 8,269)	AKI 1 (n = 1,263)	AKI 2 (n = 541)	AKI 3 (n = 354)	*P*	No AKI (n = 5,935)	AKI 1 (n = 809)	AKI 2 (n = 271)	AKI 3 (n = 188)	*P*	No Prior AKI (n = 2,738)	Prior AKI (n = 688)	*P*
Characteristics													
Age, y	60 [45-72]	73 [63-80]	72 [60-80]	69 [58-79]	<0.001	78 [71-84]	81 [74-87]	81 [74-87]	79 [70-84]	<0.001	76 [66-83]	77 [66-84]	0.09
Age ≥ 70 y	2,440 (29.5)	764 (60.5)	308 (56.9)	173 (48.9)	<0.001	4,659 (78.5)	699 (86.4)	239 (88.2)	148 (78.7)	<0.001	1,847 (67.5)	484 (70.3)	0.01
Female sex	4,609 (55.7)	621 (49.2)	276 (51.0)	160 (45.2)		3,578 (60.3)	463 (57.2)	165 (60.9)	91 (48.4)		1,414 (51.6)	362 (52.6)	
Male sex	3,660 (44.3)	642 (50.8)	265 (49.0)	194 (54.8)	<0.001	2,357 (39.7)	346 (42.8)	106 (39.1)	97 (51.6)	0.005	1,324 (48.4)	326 (47.4)	0.1
Ward													
Medical	2,990 (36.2)	439 (34.8)	211 (39.0)	121 (34.2)		2,069 (34.9)	273 (33.7)	93 (34.3)	80 (42.6)		916 (33.5)	301 (43.8)	
Care of the elderly	330 (4.0)	163 (12.9)	71 (13.1)	46 (13.0)		640 (10.8)	172 (21.3)	58 (21.4)	32 (17.0)		438 (16.0)	104 (15.1)	
Surgical	3,062 (37.0)	259 (20.5)	78 (14.4)	50 (14.1)		1,828 (30.8)	123 (15.2)	32 (11.8)	25 (13.3)		475 (17.4)	92 (13.4)	
Critical care unit	514 (6.2)	311 (24.6)	154 (28.5)	127 (35.9)		370 (6.2)	162 (20.0)	71 (26.2)	45 (23.9)		730 (26.7)	140 (20.4)	
Other	1,373 (16.6)	91 (7.2)	27 (5.0)	10 (2.8)	<0.001	1,028 (17.3)	79 (9.8)	17 (6.3)	6 (3.2)	<0.001	179 (6.5)	51 (7.4)	0.2
Charlson comorbid conditions^a^													
MI	255 (3.1)	91 (7.2)	36 (6.7)	23 (6.5)	<0.001	418 (7.0)	123 (15.2)	30 (11.1)	29 (15.4)	<0.001	201 (7.3)	131 (19.0)	0.2
CHF	155 (1.9)	100 (7.9)	36 (6.7)	27 (7.6)	<0.001	499 (8.4)	157 (19.4)	61 (22.5)	47 (25.0)	<0.001	227 (8.3)	201 (29.2)	0.4
PVD	167 (2.0)	83 (6.6)	26 (4.8)	18 (5.1)	<0.001	285 (4.8)	97 (12.0)	25 (9.2)	14 (7.4)	<0.001	175 (6.4)	89 (12.9)	0.9
CBVC	206 (2.5)	87 (6.9)	43 (7.9)	15 (4.2)	<0.001	439 (7.4)	94 (11.6)	25 (9.2)	13 (6.9)	<0.001	180 (6.6)	97 (14.1)	0.007
Dementia	65 (0.8)	30 (2.4)	11 (2.0)	12 (3.4)	<0.001	142 (2.4)	37 (4.6)	9 (3.3)	8 (4.3)	0.001	61 (2.2)	46 (6.7)	0.8
CPD	383 (4.6)	157 (12.4)	73 (13.5)	36 (10.2)	<0.001	407 (6.9)	92 (11.4)	42 (15.5)	18 (9.6)	<0.001	284 (10.4)	134 (19.5)	0.5
Rheumatic disease	140 (1.7)	34 (2.7)	22 (4.1)	14 (4.0)	<0.001	139 (2.3)	39 (4.8)	18 (6.6)	7 (3.7)	<0.001	79 (2.9)	55 (8.0)	0.1
Peptic ulcer disease	123 (1.5)	52 (4.1)	19 (3.5)	14 (4.0)	<0.001	160 (2.7)	27 (3.3)	16 (5.9)	5 (2.7)	0.02	83 (3.0)	50 (7.3)	0.7
Mild liver disease	88 (1.1)	35 (2.8)	17 (3.1)	15 (4.2)	<0.001	55 (0.9)	<5^a^ (-)	6 (2.2)	<5^a^ (-)	0.02	46 (1.7)	34 (4.9)	0.7
Severe liver disease	32 (0.4)	11 (0.9)	10 (1.8)	5 (1.4)	<0.001	13 (0.2)	<5^a^ (-)	<5^a^ (-)	<5^a^ (-)	0.3	16 (0.6)	16 (2.3)	0.07
DM without complications	301 (3.6)	125 (9.9)	58 (10.7)	43 (12.1)	<0.001	470 (7.9)	129 (15.9)	44 (16.2)	40 (21.3)	<0.001	267 (9.8)	172 (25.0)	0.7
DM with complications	15 (0.2)	19 (1.5)	9 (1.7)	7 (2.0)	<0.001	62 (1.0)	33 (4.1)	13 (4.8)	7 (3.7)	<0.001	48 (1.8)	40 (5.8)	0.9
Hemiplegia	39 (0.5)	13 (1.0)	7 (1.3)	<5^a^ (-)	0.007	34 (0.6)	9 (1.1)	<5^a^ (-)	<5^a^ (-)	0.1	18 (0.7)	20 (2.9)	0.4
Malignancy	520 (6.3)	213 (16.9)	104 (19.2)	72 (20.3)	<0.001	530 (8.9)	83 (10.3)	38 (14.0)	31 (16.5)	<0.001	420 (15.3)	121 (17.6)	0.9
Metastatic malignancy	111 (1.3)	58 (4.6)	41 (7.6)	28 (7.9)	<0.001	80 (1.3)	16 (2.0)	6 (2.2)	7 (3.7)	0.03	128 (4.7)	28 (4.1)	0.9

*Note:* The cohort includes all with abnormal kidney function and a 20% random sample of those with normal kidney function. Patients with no AKI and normal baseline function are therefore under-represented in this table. Values for categorical variables are given as number (percentage); values for continuous variables, as median [interquartile range]. *P* values are from χ^2^ test, or Kruskal-Wallis test when appropriate.

Abbreviations and definitions: AKI, acute kidney injury (1-3 denote severity stage); CBVD, cerebrovascular disease; CHF, congestive heart failure; CPD, chronic pulmonary disease; DM, diabetes mellitus; eGFR, estimated glomerular filtration rate; MI, myocardial infarction; PVD, peripheral vascular disease.

a Results for human immunodeficiency virus and small numbers not reported to prevent patient identification.

**Table 2:** Crude Mortality Outcomes up to 10 Years

Mortality	Normal Baseline Function: eGFR ≥ 60 mL/min/1.73 m^2^				Baseline eGFR 45-59 mL/min/1.73 m^2^				Baseline eGFR 30-44 mL/min/1.73 m^2^				Baseline eGFR < 30 mL/min/1.73 m^2^				Prior AKI Episodes	
	No AKI (n = 8,269)	AKI 1 (n = 1,263)	AKI 2 (n = 541)	AKI 3 (n = 354)	No AKI (n = 3,672)	AKI 1 (n = 375)	AKI 2 (n = 157)	AKI 3 (n = 72)	No AKI (n = 1,666)	AKI 1 (n = 287)	AKI 2 (n = 88)	AKI 3 (n = 49)	No AKI (n = 597)	AKI 1 (n = 147)	AKI 2 (n = 26)	AKI 3 (n = 67)	No Prior (n = 2,738)	Prior AKI (n = 688)
30 d	108 (1.3)	151 (12.0)	113 (20.9)	109 (30.8)	111 (3.0)	49 (13.1)	32 (20.4)	23 (32)	104 (6.2)	42 (14.6)	23 (26)	14 (29)	66 (11.1)	25 (17.0)	7 (27)	16 (24)	481 (17.6)	123 (17.9)
1 y	528 (6.4)	380 (30.1)	243 (44.9)	210 (59.3)	429 (11.7)	129 (34.4)	78 (49.7)	47 (65)	329 (19.7)	117 (40.8)	56 (64)	32 (65)	172 (28.8)	67 (45.6)	16 (62)	36 (54)	1,093 (39.9)	318 (46.2)
5 y	1,583 (19.1)	696 (55.1)	352 (65.1)	252 (71.2)	1,411 (38.4)	251 (66.9)	111 (70.7)	56 (78)	928 (55.7)	222 (77.4)	79 (90)	42 (86)	411 (68.8)	116 (78.9)	21 (81)	52 (78)	1,747 (63.8)	503 (73.1)
10 y	2,509 (30.3)	877 (69.4)	417 (77.1)	282 (79.7)	2,219 (60.4)	313 (83.5)	145 (92.4)	64 (89)	1,296 (77.8)	267 (93.0)	83 (94)	45 (92)	521 (87.3)	134 (91.2)	23 (89)	56 (83)	2,111 (77.1)	595 (86.5)

*Note:* The cohort includes all with abnormal kidney function and a 20% random sample of those with normal kidney function. Patients with no AKI and normal baseline are therefore under-represented in this table. Values are given as number (percentage).

Abbreviations and definitions: AKI, acute kidney injury (1-3 denote severity stage); eGFR, estimated glomerular filtration rate.

**Table 3:** Ten-Year Mortality Conditional on Surviving the First Year

	No.	Age- & Sex-Adjusted HR (95% CI)	Fully adjusted HR^a^ (95% CI)
eGFR ≥ 60, no AKI	7,741	1.00 (reference)	1.00 (reference)
eGFR ≥ 60, AKI	1,325	1.74 (1.60-1.89)	1.44 (1.31-1.58)
**AKI vs no AKI for eGFR ≥ 60 group**		**1.74 (1.60-1.89)**	**1.44 (1.31-1.58)**
eGFR 45-59, no AKI	3,243	1.11 (1.04-1.19)	1.09 (1.02-1.17)
eGFR 45-59, AKI	350	1.77 (1.56-2.02)	1.36 (1.19-1.56)
**AKI vs no AKI for eGFR 45-59 group**		**1.59 (1.40-1.81)**	**1.25 (1.09-1.43)**
eGFR 30-44, no AKI	1,337	1.48 (1.36-1.61)	1.40 (1.29-1.52)
eGFR 30-44, AKI	219	2.16 (1.85-2.52)	1.69 (1.66-2.11)
**AKI vs no AKI for eGFR 30-44 group**		**1.49 (1.25-1.70)**	**1.21 (1.03-1.42)**
eGFR < 30, no AKI	425	2.08 (1.85-2.34)	1.87 (1.66-2.11)
eGFR < 30, AKI	121	2.28 (1.85-2.81)	2.02 (1.62-2.50)
**AKI vs no AKI for eGFR < 30 group**		**1.10 (0.87-1.38)**	**1.08 (0.85-1.36)**

*Note:* Multivariable Cox regression with interaction terms between AKI and baseline eGFR. Adjusted HRs are reported with reference to no AKI and normal baseline kidney function and for baseline eGFR groups calculated using the interaction terms. eGFRs expressed in mL/min/1.73 m^2^. Boldface indicates AKI vs no AKI within each eGFR group calculated using the interaction terms.

Abbreviations: AKI, acute kidney injury; CI, confidence interval; eGFR, estimated glomerular filtration rate; HR, hazard ratio.

a Adjusted for age, sex, Charlson comorbid conditions, hospital admission circumstances, *International Classification of Diseases, Tenth Revision* categories for acute hospital admission diagnoses, and with interaction terms between AKI and baseline eGFR.

## Box 1 AKI Criteria for This Study and Definitions for AKI Severity, Prior AKI, and 7-Day Recovery

**AKI criteria definitions**^**a**^

• Modified criterion 1: Scr ≥ 1.5 times higher than median of all Scr values 8-90 d ago, or 91-365 d ago if no tests between 8-90 d
• Criterion 2: Scr ≥ 1.5 times higher than lowest Scr within 7 d
• Criterion 3: Scr > 26 μmol/L higher than lowest Scr within 48 h

**AKI severity staging definitions**^**b**^

• Stage 1: Increase in Scr of 0.3 mg/dL; or index/baseline ratio ≥1.5-<2
• Stage 2: Index/baseline ratio ≥2-<3
• Stage 3: Index/baseline ratio ≥3; or ≥1.5 and index Scr > 4 mg/dL (or 3× upper reference interval if age <18 y)

**Prior AKI episodes definition**

• No prior AKI: First AKI episode in 2003 not preceded by any previous AKI episodes in prior 91-1,095 d
• Prior AKI: First AKI episode in 2003 preceded by at least 1 previous AKI episode in prior 91-1,095 d

**7-day AKI recovery status definition^**c**^**

• Complete recovery: Last Scr within 7 d of AKI < 1.2 times higher than baseline Scr at diagnosis
• Partial recovery: Last Scr within 7 d of AKI >1.2-<1.5 times higher than baseline Scr at diagnosis
• Nonrecovery: Last Scr within 7 d of AKI ≥ 1.5 times higher than baseline Scr at diagnosis, or still receiving urgent RRT
• “Untested”: No repeat blood tests taken within 7 d of AKI diagnosis

*Note:* Conversion factor for Scr in mg/dL to μmol/L, ×88.4.

Abbreviations: AKI, acute kidney injury; RRT, renal replacement therapy; Scr, serum creatinine.

^a^One of 1-3; note criterion 1 modified from National Health Service England AKI warning criteria to enable recognition of distinct, prior, and subsequent AKI episodes.

^b^Based on peak Scr within 90 days of diagnosis.

^c^For 90-day recovery status, we applied the same thresholds but using the last available Scr within 90 days of AKI onset.

## Supplementary Material

Supplementary Table S1 (PDF) Patient characteristics by AKI severity in eGFR subgroups.

Supplementary Table S2 (PDF) Crude long-term RRT outcomes up to 10 y.

Supplementary Table S3 (PDF) Crude long-term RRT outcomes up to 10 y in eGFR subgroups.

Supplementary Table S4 (PDF) Sensitivity analyses for mortality after AKI vs no AKI.
