# Supplementary material for: Intermediate and Long-term Outcomes of Survivors of Acute Kidney Injury Episodes: A Large Population-Based Cohort Study
Source: Am J Kidney Dis. 2017 Jan;69(1):18–28. doi: 10.1053/j.ajkd.2016.05.018 (PMC5176133; doi:10.1053/j.ajkd.2016.05.018)
Supplement: Supplementary Table S1 (PDF) — Patient characteristics by AKI severity in eGFR subgroups. [file mmc1.pdf]

Table S1 – Patient characteristics by AKI severity in eGFR subgroups

	Baseline eGFR 45-59 ml/min/1.73m^2							
	No AKI		AKI 1		AKI 2		AKI 3	
n	3672		375		157		72	
**CHARACTERISTICS**								
Median age in years (IQR)	76	(69-83)	81	(73-85)	81	(73-86)	78	(78-83)
Age ≥70 years	2737	(74.5)	321	(85.6)	137	(87.3)	57	(79.2)
Female	2185	(59.5)	211	(56.3)	93	(59.2)	37	(51.4)
Male	1487	(40.5)	164	(43.7)	64	(40.8)	35	(48.6)
[a]Medical or other ward	1902	(51.8)	163	(43.5)	72	(45.9)	30	(41.7)
Care of the elderly ward	328	(8.9)	77	(20.5)	31	(19.7)	9	(12.5)
Surgical ward	1209	(32.9)	60	(16.0)	17	(10.8)	10	(13.9)
Critical care unit	233	(6.3)	75	(20.0)	37	(23.6)	23	(31.9)
**[a]CHARLSON COMORBIDITIES**								
Myocardial infarction	227	(6.2)	52	(13.9)	11	(7.0)	12	(16.7)
Congestive heart failure	224	(6.1)	56	(14.9)	24	(15.3)	19	(26.4)
Peripheral vascular disease	143	(3.9)	39	(10.4)	16	(10.2)	6	(8.3)
Cerebrovascular disease	226	(6.2)	46	(12.3)	16	(10.2)	<5[a]	(-)
Dementia	68	(1.9)	14	(3.7)	<5[a]	(-)	<5[a]	(-)
Chronic pulmonary disease	229	(6.2)	36	(9.6)	25	(15.9)	9	(12.5)
Rheumatic disease	84	(2.3)	22	(5.9)	8	(5.1)	<5[a]	(-)
Peptic ulcer disease	92	(2.5)	14	(3.7)	10	(6.4)	<5[a]	(-)
Mild liver disease	29	(0.8)	<5[a]	(-)	<5[a]	(-)	<5[a]	(-)
Severe liver disease	11	(0.3)	<5[a]	(-)	<5[a]	(-)	<5[a]	(-)
Diabetes without complications	245	(6.7)	52	(13.9)	23	(14.6)	17	(23.6)
Diabetes with complications	21	(0.6)	11	(2.9)	7	(4.5)	<5[a]	(-)
Hemiplegia	15	(0.4)	5	(1.3)	<5[a]	(-)	<5[a]	(-)
Malignancy	336	(9.2)	47	(12.5)	25	(15.9)	10	(13.9)
Metastatic malignancy	51	(1.4)	11	(2.9)	5	(3.2)	<5[a]	(-)

	Baseline eGFR 30-44 ml/min/1.73m^2							
	No AKI		AKI 1		AKI 2		AKI 3	
n	1666		287		88		49	
**CHARACTERISTICS**								
Median age in years (IQR)	81	(74-87)	82	(76-88)	83	(75-88)	81	(75-85)
Age ≥70 years	1420	(85.2)	255	(88.9)	76	(86.4)	46	(93.9)
Female	1033	(62.0)	165	(57.5)	56	(63.6)	23	(46.9)
Male	633	(38.0)	122	(42.5)	32	(36.4)	26	(53.1)
[a]Medical or other ward	855	(51.3)	123	(42.9)	29	(33.0)	24	(49.0)
Care of the elderly ward	226	(13.6)	69	(24.0)	22	(25.0)	11	(22.4)
Surgical ward	479	(28.8)	39	(13.6)	12	(13.6)	8	(16.3)
Critical care unit	106	(6.4)	56	(19.5)	25	(28.4)	6	(12.2)
**[a]CHARLSON COMORBIDITIES**								
Myocardial infarction	138	(8.3)	51	(17.8)	15	(17.0)	7	(14.3)
Congestive heart failure	181	(10.9)	68	(23.7)	27	(30.7)	13	(26.5)
Peripheral vascular disease	97	(5.8)	44	(15.3)	8	(9.1)	<5[a]	(-)
Cerebrovascular disease	147	(8.8)	34	(11.8)	6	(6.8)	5	(10.2)
Dementia	46	(2.8)	15	(5.2)	6	(6.8)	<5[a]	(-)
Chronic pulmonary disease	128	(7.7)	45	(15.7)	13	(14.8)	<5[a]	(-)
Rheumatic disease	40	(2.4)	14	(4.9)	9	(10.2)	<5[a]	(-)
Peptic ulcer disease	48	(2.9)	11	(3.8)	5	(5.7)	<5[a]	(-)
Mild liver disease	18	(1.1)	<5[a]	(-)	<5[a]	(-)	<5[a]	(-)
Severe liver disease	<5[a]	(-)	<5[a]	(-)	<5[a]	(-)	<5[a]	(-)
Diabetes without complications	145	(8.7)	54	(18.8)	16	(18.2)	8	(16.3)
Diabetes with complications	23	(1.4)	15	(5.2)	6	(6.8)	<5[a]	(-)
Hemiplegia	14	(0.8)	<5[a]	(-)	<5[a]	(-)	<5[a]	(-)
Malignancy	144	(8.6)	24	(8.4)	13	(14.8)	7	(14.3)
Metastatic malignancy	23	(1.4)	<5[a]	(-)	<5[a]	(-)	<5[a]	(-)

	Baseline eGFR <30ml/min/1.73m^2							
	No AKI		AKI 1		AKI 2		AKI 3	
n	597		147		26		67	
**CHARACTERISTICS**								
Median age in years (IQR)	82	(75-88)	81	(75-87)	82	(75-87)	77	(65-84)
Age ≥70 years	502	(84.1)	123	(83.7)	23	(88.5)	45	(67.2)
Female	360	(60.3)	87	(59.2)	16	(61.5)	31	(46.3)
Male	237	(39.7)	60	(40.8)	10	(38.5)	36	(53.7)
[a]Medical or other ward	231	(38.7)	66	(44.9)	9	(34.6)	32	(47.8)
Care of the elderly ward	86	(14.4)	26	(17.7)	5	(19.2)	12	(17.9)
Surgical ward	140	(23.5)	24	(16.3)	<5[a]	(-)	7	(10.4)
Critical care unit	140	(23.5)	31	(21.1)	9	(34.6)	16	(23.9)
**[a]CHARLSON COMORBIDITIES**								
Myocardial infarction	53	(8.9)	20	(13.6)	<5[a]	(-)	10	(14.9)
Congestive heart failure	94	(15.7)	33	(22.4)	10	(38.5)	15	(22.4)
Peripheral vascular disease	45	(7.5)	14	(9.5)	<5[a]	(-)	<5[a]	(-)
Cerebrovascular disease	66	(11.1)	14	(9.5)	<5[a]	(-)	6	(9.0)
Dementia	28	(4.7)	8	(5.4)	<5[a]	(-)	<5[a]	(-)
Chronic pulmonary disease	50	(8.4)	11	(7.5)	<5[a]	(-)	5	(7.5)
Rheumatic disease	15	(2.5)	<5[a]	(-)	<5[a]	(-)	<5[a]	(-)
Peptic ulcer disease	20	(3.4)	<5[a]	(-)	<5[a]	(-)	<5[a]	(-)
Mild liver disease	8	(1.3)	<5[a]	(-)	<5[a]	(-)	<5[a]	(-)
Severe liver disease	<5[a]	(-)	<5[a]	(-)	<5[a]	(-)	<5[a]	(-)
Diabetes without complications	80	(13.4)	23	(15.6)	5	(19.2)	15	(22.4)
Diabetes with complications	18	(3.0)	7	(4.8)	<5[a]	(-)	<5[a]	(-)
Hemiplegia	7	(1.2)	<5[a]	(-)	<5[a]	(-)	<5[a]	(-)
Malignancy	50	(8.4)	12	(8.2)	<5[a]	(-)	14	(20.9)
Metastatic malignancy	6	(1.0)	<5[a]	(-)	<5[a]	(-)	<5[a]	(-)

Abbreviations: AKI, acute kidney injury (1-3 denote severity stage); eGFR, estimated glomerular filtration rate

[a]Results for human immunodeficiency virus, "other" ward location, and small numbers not reported to prevent patient identification at the request of clinical lead for Grampian Data Safe-Haven
