# Supplementary material for: Intermediate and Long-term Outcomes of Survivors of Acute Kidney Injury Episodes: A Large Population-Based Cohort Study
Source: Am J Kidney Dis. 2017 Jan;69(1):18–28. doi: 10.1053/j.ajkd.2016.05.018 (PMC5176133; doi:10.1053/j.ajkd.2016.05.018)
Supplement: Supplementary Table S2 (PDF) — Crude long-term RRT outcomes up to 10 y. [file mmc2.pdf]

Table S2 – Crude long-term RRT outcomes up to 10 years

	**Normal baseline (eGFR ≥60 ml/min/1.73m²)**								**Baseline decreased kidney function (eGFR <60 ml/min/1.73m²)**								**Prior AKI episodes**			
	**No AKI**		**AKI 1**		**AKI 2**		**AKI 3**		**No AKI**		**AKI 1**		**AKI 2**		**AKI 3**		**No prior AKI**		**Prior AKI**	
n	8269		1263		541		354		5935		809		271		188		2738		688	
Long-term RRT																				
1 year									23	(0.4)	7	(0.9)	2	(0.8)	22	(11.7)	17	(0.6)	14	(2.0)
5 years									63	(1.1)	21	(2.6)	4	(1.5)	24	(12.8)	32	(1.2)	18	(2.6)
10 years	[a]10	(0.1)			[a]6	(0.3)			86	(1.4)	26	(3.2)	5	(1.8)	28	(14.9)	44	(1.6)	21	(3.1)

Note: The cohort includes all with abnormal kidney function and a 20% random sample of those with normal kidney function. Patients with no AKI and normal baseline are therefore under-represented in this table. Figures in brackets denote percentages.

Abbreviations: AKI, acute kidney injury (1-3 denote severity stage); eGFR, estimated glomerular filtration rate; RRT, renal replacement therapy

[a]Applies to all no AKI and AKI patients with normal baseline, small numbers do not allow AKI subgroups to be reported to prevent patient identification
