# Supplementary material for: Intermediate and Long-term Outcomes of Survivors of Acute Kidney Injury Episodes: A Large Population-Based Cohort Study
Source: Am J Kidney Dis. 2017 Jan;69(1):18–28. doi: 10.1053/j.ajkd.2016.05.018 (PMC5176133; doi:10.1053/j.ajkd.2016.05.018)
Supplement: Supplementary Table S3 (PDF) — Crude long-term RRT outcomes up to 10 y in eGFR subgroups. [file mmc3.pdf]

Table S3 – Crude long-term RRT outcomes up to 10 years in eGFR subgroups

	Normal baseline (eGFR ≥60ml/min/1.73m²)				Baseline eGFR 45-59 ml/min/1.73m²				Baseline eGFR 30-44 ml/min/1.73m²				Baseline eGFR <30ml/min/1.73m²			
	No AKI	AKI 1	AKI 2	AKI 3	No AKI	AKI 1	AKI 2	AKI 3	No AKI	AKI 1	AKI 2	AKI 3	No AKI	AKI 1	AKI 2	AKI 3
n	8269	1263	541	354	3672	375	157	72	1666	287	88	49	597	147	26	67
Long-term RRT																
1 year	5 (0.1)	<3 (-)	<3 (-)	<3 (-)	<3 (-)	<3 (-)	<3 (-)	<3 (-)	<3 (-)	<3 (-)	<3 (-)	5 (10.2)	22 (3.7)	6 (4.1)	<3 (-)	15 (22.4)
5 years	10 (0.1)	<3 (-)	<3 (-)	<3 (-)	4 (0.1)	3 (0.8)	<3 (-)	3 (4.2)	11 (0.7)	3 (1.0)	<3 (-)	5 (10.2)	48 (8.0)	15 (10.2)	3 (11.5)	16 (23.9)
10 years	10 (0.1)	<3 (-)	4 (0.7)	<3 (-)	8 (0.2)	6 (1.6)	<3 (-)	3 (4.2)	21 (1.3)	3 (1.0)	<3 (-)	6 (12.2)	57 (9.5)	17 (11.6)	3 (11.5)	19 (28.4)

Note: The cohort includes all with abnormal kidney function and a 20% random sample of those with normal kidney function. Patients with no AKI and normal baseline are therefore under-represented in this table. Figures in brackets denote percentages. Small numbers have been suppressed to avoid patient identification

Abbreviations: AKI, acute kidney injury (1-3 denote severity stage); eGFR, estimated glomerular filtration rate; RRT, renal replacement therapy
