# Supplementary material for: Intermediate and Long-term Outcomes of Survivors of Acute Kidney Injury Episodes: A Large Population-Based Cohort Study
Source: Am J Kidney Dis. 2017 Jan;69(1):18–28. doi: 10.1053/j.ajkd.2016.05.018 (PMC5176133; doi:10.1053/j.ajkd.2016.05.018)
Supplement: Supplementary Table S4 (PDF) — Sensitivity analyses for mortality after AKI vs no AKI. [file mmc4.pdf]

Table S4 – Sensitivity analyses for mortality after AKI vs no AKI (multivariable Cox regression)

	Intermediate mortality (30-365 days)			Long-term mortality (1-10 years) limited to those aged <75 years			Long-term mortality (1-10 years) limited to AKI without complete recovery			Long-term mortality (1-10 years) limited to AKI with prior AKI episodes		
	**N**	**Age-sex adjusted**	**Fully adjusted[a]**	**N**	**Age-sex adjusted**	**Fully adjusted[a]**	**N**	**Age-sex adjusted**	**Fully adjusted[a]**	**N**	**Age-sex adjusted**	**Fully adjusted[a]**
eGFR ≥ 60, no AKI (reference)	8161	1.0	1.0	6461	1.0	1.0	7741	1.0	1.0	7741	1.0	1.0
eGFR ≥ 60, AKI	1785	3.70 (3.23-4.24)	2.48 (2.15-2.88)	855	2.19 (1.94-2.47)	1.50 (1.31-1.72)	683	1.71 (1.53-1.90)	1.42 (1.26-1.59)	198	2.45 (2.05-2.93)	1.64 (1.36-1.97)
**AKI vs no AKI for eGFR group**		**3.70 (3.23-4.24)**	**2.48 (2.15-2.88)**		**2.19 (1.94-2.47)**	**1.50 (1.31-1.72)**		**1.71 (1.53-1.90)**	**1.42 (1.26-1.59)**		**2.45 (2.05-2.93)**	**1.64 (1.36-1.97)**
eGFR 45-59, no AKI	3561	0.90 (0.77-1.05)	0.90 (0.77-1.04)	1475	1.24 (1.11-1.38)	1.18 (1.05-1.32)	3243	1.09 (1.02-1.17)	1.07 (1.00-1.14)	3243	1.09 (1.02-1.17)	1.07 (1.00-1.15)
eGFR 45-59, AKI	500	3.12 (2.57-3.79)	2.24 (1.83-2.74)	123	2.30 (1.80-2.94)	1.67 (1.29-2.15)	133	1.88 (1.54-2.29)	1.42 (1.16-1.75)	78	2.10 (1.64-2.70)	1.18 (0.91-1.53)
**AKI vs no AKI for eGFR group**		**3.47 (2.85-4.21)**	**2.50 (2.04-3.06)**		**1.86 (1.45-2.39)**	**1.41 (1.09-1.83)**		**1.72 (1.41-2.09)**	**1.33 (1.09-1.63)**		**1.92 (1.50-2.46)**	**1.10 (0.85-1.43)**
eGFR 30-44, no AKI	1562	1.25 (1.06-1.49)	1.20 (1.01-1.43)	383	1.94 (1.65-2.28)	1.60 (1.35-1.89)	1337	1.44 (1.33-1.57)	1.36 (1.25-1.48)	1337	1.45 (1.33-1.57)	1.36 (1.25-1.48)
eGFR 30-44, AKI	345	3.44 (2.79-4.24)	2.29 (1.84-2.86)	56	3.72 (2.70-5.12)	2.52 (1.80-3.53)	72	2.28 (1.78-2.92)	1.68 (1.30-2.17)	57	3.00 (2.27-3.96)	1.52 (1.14-2.03)
**AKI vs no AKI for eGFR group**		**2.74 (2.20-3.41)**	**1.90 (1.51-2.39)**		**1.92 (1.36-2.71)**	**1.58 (1.10-2.25)**		**1.58 (1.23-2.02)**	**1.24 (0.96-1.60)**		**2.07 (1.56-2.73)**	**1.12 (0.83-1.49)**
eGFR < 30, no AKI	531	1.75 (1.40-2.18)	1.57 (1.26-1.97)	129	3.36 (2.67-4.24)	2.53 (1.98-3.22)	425	2.03 (1.80-2.29)	1.80 (1.60-2.04)	425	2.04 (1.81-2.30)	1.81 (1.61-2.05)
eGFR < 30, AKI	192	4.09 (3.16-5.29)	2.57 (1.95-3.37)	46	3.09 (2.06-4.64)	2.73 (1.79-4.17)	35	2.49 (1.70-3.64)	2.27 (1.54-3.34)	37	2.29 (1.61-3.27)	1.71 (1.18-2.47)
**AKI vs no AKI for eGFR group**		**2.33 (1.73-3.16)**	**1.63 (1.20-2.22)**		**0.92 (0.58-1.45)**	**1.08 (0.68-1.72)**		**1.23 (0.83-1.82)**	**1.26 (0.84-1.87)**		**1.12 (0.78-1.62)**	**0.94 (0.65-1.37)**

Note: Adjusted hazard ratios are reported with reference to no AKI and normal baseline function (plain type); and for baseline eGFR groups calculated using the interaction terms (bold type). 95% confidence intervals are reported in brackets.
Abbreviations: AKI, acute kidney injury; eGFR, estimated glomerular filtration rate.
[a]Adjusted for age, sex, Charlson comorbidities, hospital admission circumstances, ICD-10 categories for acute hospital admission diagnoses, and with interaction terms between AKI and baseline eGFR.
